# *Mycobacterium tuberculosis* Diversity Exploration: A Way to Serve the Three Main Weapons against Epidemics, Hygiene, Vaccine Development and Chemotherapy

**DOI:** 10.3390/microorganisms10081492

**Published:** 2022-07-25

**Authors:** Guislaine Refrégier, Charlotte Genestet

**Affiliations:** 1 Université Paris-Saclay, CNRS, AgroParisTech, Ecologie Systématique et Evolution, 91190 Gif-sur-Yvette, France; 2 Laboratoire de Bactériologie, Institut des Agents Infectieux, Hospices Civils de Lyon, CEDEX 04, 69317 Lyon, France; charlotte.genestet@gmail.com; 3 CIRI—Centre International de Recherche en Infectiologie, Ecole Normale Supérieure de Lyon, Université Claude Bernard Lyon 1, Inserm U1111, CNRS UMR5308, 69007 Lyon, France

As highlighted by the SARS pandemic which is still ongoing, the battle against pathogens relies on three main “weapons”: hygiene, vaccine development and chemotherapy strategies. All of these strategies benefit from a deep understanding of the diversity and physiology of the pathogen and of its interactions with the host [1,2,3,4]. Indeed, molecular epidemiology helps to refine hygiene measures by tracking where transmission occurs, efficient vaccines are often derived from a good understanding of the molecules exposed at the pathogen surface, and chemotherapy can be diversified through an in-depth understanding of key physiological mechanisms such as important metabolic pathways. Depending on the pathogen, the use of just one of these weapons may prove sufficient to effectively reduce the use of others. For example, COVID is mainly handled using vaccination and hygiene measures [5], and smallpox was eradicated thanks to vaccination [6]. However, to fight a tenacious pathogen, all of these weapons should certainly be considered and finely tuned.

Here, we are interested in tuberculosis (TB). TB remains one of the most prevalent and deadly infectious diseases, causing death in around 1.3 million HIV-negative people in 2020 (WHO, Geneva, Switzerland, 2021). TB is caused by the bacteria referred to as the *Mycobacterium tuberculosis* complex (Mtbc). Intriguingly, most people infected by *M. tuberculosis* do not present symptoms. They contain the bacillus without eradicating it; in other words, they exhibit latent tuberculosis. In contrast, people with decreased immunity due to bad health conditions or comorbidities reach an active state of the disease. Latent tuberculosis was estimated to affect around 23% of the population in 2014 [7]. This suggests that today, around 1.8 billion people are hosting this bacterium. This reservoir is a time bomb: TB can widely increase its transmission rate if hygiene levels decrease. It is therefore a major public health concern. Accordingly, TB is cited together with AIDS and malaria in the 3rd goal of Sustainable Development Goals 2030 agenda (https://sdgs.un.org/2030agenda (accessed on 20 July 2022)). This specific goal consists of ending TB by 2030, i.e., reducing the incidence by 80% and deaths by 90%. However, the road to ending the prevalence of TB and, even more so, to eradicating TB could be longer, especially considering delayed diagnostics and treatment onsets related to the COVID pandemic in 2020 (WHO, Geneva, Switzerland, 2021). To try to meet this goal, all efforts are definitively required. 

As mentioned above, several weapons may be used to manage and eradicate TB: hygiene, vaccination and chemotherapy. These are all weapons that, to be refined, require sharp understanding of the molecular mechanisms at play. This is especially true for a pathogen that, if not very diverse, is at least very versatile: Mtbc infections result in a wide spectrum of clinical outcomes, from latent asymptomatic infection to pulmonary or extra- pulmonary manifestations of disease, with diversity in the severity of symptoms [8,9]. Such diversity has been historically attributed to host and environmental factors, and bacterial diversity was considered to be of limited interest in our understanding of the disease [10]. Thus, bacterial diversity firstly explored to track epidemics. Limited typing and Whole-Genome Sequencing have identified six major and three additional rare lineages of human-adapted Mtbc, which epidemiologically co-associate with distinct geographical regions [11,12]. The impact of this macrodiversity on disease features (host adaptation and transmissibility) has received ever-growing support in the last ten years: from the disruption of the sympatric host–pathogen interactions in human tuberculosis [13] to the identification of sublineages significantly associated with severe symptoms [14]. 

In this Special Issue, we aimed to make all approaches visible, contributing to a better understanding of micro- and macrodiversity in *M. tuberculosis* and its impact. This includes the presentation of new methods to describe diversity, from the exploration of DNA diversity to metabolic diversity, and from wide-scale exploration to microdiversity dissection. This Special Issue seems timely in the sense that new technologies and the application of these new technologies to complex systems, such as long-term host–pathogen interactions, have been democratized. Studies have recently taken place in various settings, including regions that have long had little means with which to carry out research. These studies shed new light on the actual genetic diversity of pathogens and of the interplay between bacterial genetics and disease symptoms, ans this is particularly true for TB. By bringing these studies together, we hope to help the reader develop a richer understanding of the complexity at play in the battle against TB.
